# Importance of Promyelocytic Leukema Protein (PML) for Kaposi’s Sarcoma-Associated Herpesvirus Lytic Replication

**DOI:** 10.3389/fmicb.2018.02324

**Published:** 2018-10-08

**Authors:** Md. Golzar Hossain, Eriko Ohsaki, Tomoyuki Honda, Keiji Ueda

**Affiliations:** Division of Virology, Department of Microbiology and Immunology, Graduate School of Medicine, Osaka University, Osaka, Japan

**Keywords:** Kaposi’s sarcoma-associated herpesvirus (KSHV), latency, lytic replication, promyelocytic leukema protein (PML), ND10, PEL cells

## Abstract

Many DNA virus replication-related proteins are associated with promyelocytic leukemia protein (PML), a component of nuclear domain 10 (ND10), which has been investigated for its potential involvement in viral replication. In the case of Kaposi’s sarcoma-associated herpesvirus (KSHV) lytic gene products, K8 (K-bZIP), ORF59, and ORF75 have been shown to colocalize with PML, but its importance in KSHV lytic replication is still unclear. In this study, we analyzed the functional influence of PML on KSHV latency and lytic replication in KSHV-infected primary effusion lymphoma (PEL) cell lines. Stable PML-knockout (BC3-PML^KO^) and PML-overexpressing BC3 cells (BC3^PML^) were successfully generated and the latency and reactivation status were analyzed. The results demonstrated that neither KSHV latency nor the episome copy number was affected in BC3-PML^KO^ cells. In the reactivation phase, the expression dynamics of KSHV immediate-early or early lytic proteins such as RTA, K9 (vIRF1), K5, K3, ORF59, and K8 (K-bZIP) were comparable between wild-type, control BC3, and BC3-PML^KO^ cells. Interestingly, KSHV lytic replication, virion production, and expression of late genes were downregulated in BC3-PML^KO^ cells and upregulated in BC3^PML^ cells, compared to those in control or wild-type BC3 cells. Moreover, exogenous PML increased the size of the PML dots and recruited additional K8 (K-bZIP) to PML-NBs as dots. Therefore, PML would function as a positive regulator for KSHV lytic DNA replication by recruiting KSHV replication factors such as 8 (K-bZIP) or ORF59 to the PML-NBs.

## Introduction

Kaposi’s sarcoma-associated herpesvirus (KSHV), also called human herpesvirus 8 (HHV-8), is a large double-stranded DNA virus belonging to the herpesviridae family and γ-herpesvirinae subfamily (Wen and Damania, 2010). The KSHV genome size is approximately 165 kb and the nucleocapsid is surrounded by a tegument layer which lies beneath the envelope layer (Sathish et al., 2012). The KSHV genome encodes more than 80 viral open reading frames (ORFs) and 12 microRNAs (Jenner et al., 2001; Umbach and Cullen, 2010; Qin et al., 2014).

Kaposi’s sarcoma-associated herpesvirus was identified from Kaposi’s sarcoma (KS) tissues of patients with acquired immunodeficiency syndrome (AIDS) and was confirmed to be an etiological agent for the development of KS (Chang et al., 1994; Cesarman et al., 1995). It is also involved in two important lymphoproliferative disorders, primary effusion lymphoma (PEL) and multicentric Castleman’s disease (MCD) (Cesarman and Knowles, 1999). Like the other herpesviruses, KSHV has two modes of infection: latency and lytic replication (Lee et al., 2010; Uppal et al., 2014). The latency is regulated mainly by latency-associated nuclear antigen (LANA), which is one of the most important proteins (Rainbow et al., 1997). During latent infection, the virus expresses a few genes along with LANA, such as *kaposin, viral cyclin D* (*v-Cyc/ORF72*), *viral FLICE inhibitory* protein (*v-FLIP), K13/ORF71, miRNAs*, and *vIRF3* (McCormick and Ganem, 2005; Lee et al., 2010; Wen and Damania, 2010).

The latent phase may switch to the lytic phase in response to certain signals, leading to activation of the replication and transcription activator (RTA), which is a master regulator of the KSHV lytic replication (Ye et al., 2011). RTA is an immediate-early lytic protein expressed during the lytic replication, and transactivates the expression of other early and late lytic genes such as *mta (ORF57), K-bZIP (ORFK8*), *K3, K5, viral interleukin-6* (*vIL-6*), *viral interferon regulatory protein-1* (*vIRF1/ORFK9*), *ORFK1, ORF45*, and a capsid protein (*ORF65*), and concomitantly produces daughter viruses (Ueda et al., 2002; Lukac and Yuan, 2007). The *RTA* can self-activate its promoter after activation by treatment with 12-o-tetradecanoylphorbol-13-acetate (TPA) or sodium butyrate (NaB), 5-Azacytidine (5-AzaC) or trichostatin A (TSA) in PEL cells latently infected with KSHV (Chen et al., 2001; Lukac and Yuan, 2007; Li et al., 2014). During the latent phase or lytic replication, KSHV gene products interact and/or recruit and/or make complexes with many host cellular factors to maintain the latency and/or complete the lytic replication, and these involvements with host factors are likely the cause of the associated diseases. (Salsman et al., 2008; Ye et al., 2011; Li et al., 2014; Gillen et al., 2015).

Promyelocytic leukemia protein (PML), a component of nuclear domain 10 (ND10), PML oncogenic domain (POD) or PML nuclear bodies (PML-NB), has tumor suppressive and antiviral defense activities. The mammalian cells express PML in the nucleus as discrete dots varying in number (1–30 dots per nucleus) depending on the cell type, cell cycle or differentiation stage (Bernardi and Pandolfi, 2007; Geoffroy and Chelbi-Alix, 2011). Several isoforms of endogenous PML (I-VII) are generated by alternative splicing of nine major exons of the single PML gene and share the common N-terminal RBCC/tripartite motif (Exons 1–3) with varied C-terminal regions by alternative splicing of C-terminal exons. The molecular weight of the isoforms varies from 48 to 97 kD, and each is responsible for a specific function (Jensen et al., 2001; Bernardi and Pandolfi, 2007; Nisole et al., 2013). Though most of the PML isoforms are predominantly localized in the nucleus due to the presence of a nuclear localization signal in exon 6, cytoplasm-localized isoforms are also reported (Flenghi et al., 1995; Jensen et al., 2001).

Multiple post-translational modifications have been identified in the PML proteins, including sumoylation, phosphorylation, ubiquitination, and acetylation. Three major sumoylation sites identified in PML, K65, K160, and K490 are located in the RING finger, the B1 box and the nuclear localization signal (NLS), respectively, and it was reported that sumoylation of PML was necessary for the formation of PML-NB (Kamitani et al., 1998; Geoffroy and Chelbi-Alix, 2011; Nisole et al., 2013).

Promyelocytic leukemia plays an important role in the host defense mechanism as a component of innate immunity and has antiviral activities (Geoffroy and Chelbi-Alix, 2011; Lunardi et al., 2011). PML-NB interacts with various viral proteins including herpesviruses and exerts an IFN-mediated antiviral response (Everett, 2001; Ullman and Hearing, 2008; Geoffroy and Chelbi-Alix, 2011). Many studies have reported that the herpes simplex virus-1 (HSV-1) latent and lytic replication regulatory protein, ICP0 and its homologs of alpha herpesviruses such as varicella zoster virus, pseudorabies virus, and equine herpesvirus interacted and disrupted the PML with varying efficiencies during their lytic replications (Everett, 2001).

In regard to the role of PML in the KSHV replication cycle, a few facts have been revealed. Some of the recent studies showed that PML interacted with several viral proteins of KSHV during the lytic replication. The KSHV K8 (K-bZIP) protein binds with cellular p53 and colocalizes with PML-NBs, but does not disrupt PML (Katano et al., 2001). KSHV viral interferon regulatory factor 3 (vIRF3 or LANA2) increases SUMO2-ubiquitin-modified PML and disrupts the POD by a proteasome-mediated pathway, which leads to upregulation of survivin and PEL progression (Xu et al., 2004; Marcos-Villar et al., 2009). Moreover, KSHV RTA contains a SUMO-interacting motif (SIM) and acts as a SUMO-targeting ubiquitin ligase. RTA degrades SUMOs (SUMO-2/3), sumoylated PML, and K8 (K-bZIP) (Izumiya et al., 2013). However, PML-NBs are thought to be a repository of sumoylated proteins that regulate herpesvirus infection (Van Damme et al., 2010; Izumiya et al., 2013). Thus, whether the PML-NBs inhibit or enhance KSHV viral replication remains to be clarified (Saffert and Kalejta, 2008).

In this study, we generated a PML-knockout line using a KSHV-positive PEL cell line (BC3) and analyzed the functional effect of PML on KSHV latent and lytic replication. Our results demonstrated that neither the absence of PML nor the presence of exogenous PML interfered with the maintenance of KSHV latency or the expression dynamics of immediate-early or early genes after lytic induction in BC3 cells. In contrast, KSHV lytic DNA replication, extracellular virion production, and late gene expression were downregulated by PML knockout, but upregulated by exogenous PML. Moreover, exogenous PML seemed to recruit additional K8 (K-bZIP) to PML-NBs as the larger dots, and the colocalization of PML/K-bZIP dots increased significantly even though the K8 (K-bZIP) expression kinetics was not affected by PML. Thus, PML might play positive functional roles on KSHV lytic replication.

## Materials and Methods

### Cell Lines

A KSHV-infected (KSHV +) primary effusion (body cavity-based) lymphoma cell line, BC3, was grown in RPMI 1640 medium (Nacalai Tesque, Japan) containing 10% heat inactivated fetal bovine serum (FBS; Equitech-Bio, United States). The stable control (BC3-Scrambled^KO^) and PML-knockout BC3 cells (BC3-PML^KO^) were also grown in the same medium supplemented with puromycin (5 μg/mL). BC3 cells stably overexpressing PML (BC3^PML^) were established by transduction with the retrovirus expressing the longest PML (see below) and maintained in the same medium supplemented with hygromycin B (500 μg/mL). The Lenti-X 293T and GP2 cell lines (originated from HEK293) were maintained in Dulbecco’s Modified Eagle’s Medium (DMEM) with high glucose (4.5 g/L; Nacalai Tesque, Japan), supplemented with 10% Tet system approved FBS (Clontech, United States) and heat inactivated 10% FBS (Equitech-Bio, United States), respectively. All the media contained 100 μ/mL penicillin G, 100 μg/mL streptomycin, and 0.25 μg/mL amphotericin B (Nakalai Tesque, Japan), and the cells were cultured at 37°C in a 5% CO_2_ atmosphere.

### Antibodies and Reagents

A mouse monoclonal (PG-M3) and rabbit polyclonal antibodies (Ab; H-238) against PML were purchased from Santa Cruz Biotechnology and used to detect endogenous and exogenous PML. Rabbit polyclonal anti-hDAXX (HPA008736) and anti-SP100 (HPA016707) Abs were purchased from Sigma-Aldrich. Mouse monoclonal antibodies to RTA, K8 (K-bZIP), K5, K3, K9 (vIRF1), ORF59, and K8.1 were developed previously in our laboratory and rabbit polyclonal Ab against gB was a kind gift from Bala Chandran (Akula et al., 2001; Okuno et al., 2002). A rat monoclonal anti-LANA (HHV-8 ORF73) Ab was purchased from Advanced Biotechnologies (Cat# 13-210-100). An anti-Halo Tag^®^ mouse monoclonal Ab (Cat# G9211, Promega, United States) was used to detect exogenous Halo-tagged PML. A mouse monoclonal antibody (Sigma Cat# T5201) was used to detect β-tubulin. Horseradish peroxidase (HRP)-labeled goat polyclonal anti-rabbit, anti-mouse, and anti-rat immunoglobulins (Dako, Denmark) were used as secondary antibodies in Western blot analysis. Anti-mouse, anti-rat, and anti-rabbit IgG conjugated Alexa Fluor^®^ 488 and/or Alexa Fluor^®^ 546 (Molecular Probes, United States) were used as secondary antibodies for immunofluorescence assays (IFA). Tetradecanoyl phorbol acetate (TPA; Sigma, Japan) and sodium butyrate (NaB; Sigma, Japan) were used to induce lytic replication of KSHV.

### Establishment of PML-Knockout (KO) Cells

A PML sgRNA CRISPR/Cas9 All-in-One^TM^ Lentivector set (Human) purchased from (Cat# K1673405; Applied Biological Materials Inc., Canada) was used to establish PML KO cells. Three sgRNA sequences, 546 AGA UGU UGU UGG UCU UG, 666 CGC ACU UGA GCU CAC UG, and 1464 CAU CAA GAU GGA GUC UG, were designed for each vector. Lentiviruses containing such PML sgRNAs and a CRISPR/Cas9 system were produced in the Lenti-X 293T cells using a lentivirus production kit (Xfect^TM^ Transfection Reagent, Cat# 631318; Clontech, United States) according to manufacturer’s guidelines. Briefly, the Lenti-X 293T cells were plated onto (4.5 × 10^6^/100 mm dish) collagen-coated dishes (Iwaki Glass Co., Indonesia) and incubated for 24 h. Then, the medium was replaced with fresh one and transfected with the PML sgRNA CRISPR/Cas9 vectors. After 4–8 h of transfection, the medium was again replaced with fresh 10% Tet system approved FBS (Clontech, United States) in DMEM and incubated for 48 h. Then, the supernatant was passed through a 0.45 μm filter (Millex^TM^) to remove the cellular debris and the viruses encoding the three different sgRNAs were mixed together. The viral supernatant was mixed with 30% PEG8000 (PEG8000; Wako Pure Chemical Industries, United States; w/v) stock solution (final concentration, 6%) and incubated overnight at 4°C. The mixture was then centrifuged at 8400 × *g* for 30 min at 4°C and the supernatant was discarded completely. The viral pellet was dissolved in 10% FBS (in RPMI) and used for transduction to BC3 cells. The transduction experiment was performed using 48-well plates while maintaining a multiplicity of infection (MOI) of around 10. After 24 h of contact, the cells were transferred into a 25 cm^2^ flask and incubated until confluent growth of the cells. The cells were then selected with puromycin (5 μg/mL). We established two monoclonal PML-knockout cell lines of BC3 (BC3-PML^KO^#1 and BC3-PML^KO^#2) by limited dilution. As a control line, scrambled sgRNA (5′-GCA CUC ACA UCG CUA CAU CA-3′) containing All-in-One^TM^ lentiviral vector (Cat# K010, Applied Biological Materials Inc., Canada) was transduced and a polyclonal line of BC3-Scrambled ^KO^ was established. After establishing these cell lines, the cell lines were kept in the medium without puromycin.

### Establishment of Cells Stably Overexpressing PML

The full-length PML ORF tagged with a halo-tag sequence at the N-terminus was purchased from Kazusa DNA Res. Inst. (Product ID: FHC01397) and the halo-tag PML fragment was cloned into the retroviral expression vector pQCXIH (Takara-Clontech) to generate pQCXIH Halo-PML. The GP2 cells (Takara-Clontech) were used to produce a retrovirus encoding Halo-PML following the manufacturers’ manuals. Briefly, cells were seeded on 100 mm collagen-coated dishes and incubated overnight. Then, the cells were co-transfected with pQCXIH Halo-PML and pVSV-G (Clontech, United States) and the medium was changed for fresh 10% FBS in DMEM after 8 h. After 72 h of incubation, the supernatants containing infectious retroviral particles were collected and passed through a 0.45 μm filter (Millex^®^) to remove the cellular debris. The wild-type BC3 cells were transduced with the virus solution and incubated 24 h. The medium was replaced and incubated an additional 48 h. The cells were then selected with hygromycin B (Wako Pure Chemicals, Tokyo, Japan) at 500 μg/mL to isolate the cells stably expressing Halo-PML (BC3^PML^).

### Cell Viability Assay

Cell viability was determined by using the Trypan blue exclusion method. Equal amount of cell suspension and 0.4% trypan blue dye were mixed and the viable cell number was counted by a TC20 automated cell counter (Bio-Rad, United Staes). Each counting was performed in triplicate and the average value was used for the analysis.

### Lytic Induction

Wild-type (BC3), control (BC3-Scrambled^KO^), PML-knockout (BC3/PML^KO^#1 and BC3/PML^KO^#2), and PML-overexpressing (BC3^PML^) BC3 cells in a 75 cm^2^ flask (cell density, 2.5 × 10^5^ cells/mL) were treated with TPA (25 ng/ml) and NaB (0.3 mM or 0.6 mM). The cells and the culture supernatant were collected at 0, 24, 48, and 72 h after induction.

### Immunoblot Analysis

Cells were harvested by centrifugation at 180 × *g* for 5 min at 4°C. The pellet was washed with phosphate buffered saline (PBS) and lyzed with 50 mM NaH_2_PO_4_ [pH: 8.0], 300 mM NaCl, 0.1% NP40, and a complete mammalian protease inhibitor (Sigma P8849; 1:1000 dilution) to extract total protein. Immunoblotting was performed as described previously (Hossain and Ueda, 2017). The blotted PVDF membrane was blocked with 5% dry milk and cellular or viral proteins were probed with specific antibodies followed by horseradish peroxidase (HRP) conjugated immunoglobulins as secondary antibodies. Then, the chemiluminescence was visualized with ChemiDoc^®^ (Bio-Rad, United States).

### Immunofluorescence Assay

Immunofluorescence assay (IFA**)** was performed according to the previously described method (Ohsaki et al., 2004). Briefly, cells were harvested and washed two times with PBS. Then the cells were suspended in PBS, flooded onto a 24-well microscopic glass slide (Matsunami, United States) and air dried. Cells on the slide were fixed with 4% paraformaldehyde in PBS (-) at 4°C for at least 1 h and permeabilized with 0.1% Triton X-100 in PBS at room temperature for 30 min. The cells were then incubated overnight at room temperature with the respective specific primary antibody diluted in PBS containing 0.1% bovine serum albumin (BSA) and 0.02% sodium azide. After washing three times with PBS containing 0.1% Tween 20^®^ (PBS-T), the cells were incubated with fluorescent labeled secondary antibodies for at least 3 h at room temperature. Finally, the cells were stained with DAPI and mounted with glycerol for confocal microscopy analysis. The images were obtained by using a Leica TCS SP8^®^ confocal microscope, with 488 nm and 546 nm laser lines.

### DNA Extraction and Quantitative Real-Time PCR (qPCR)

Culture medium from induced or uninduced cells was collected by centrifugation at 180 × *g* for 5 min and then passed through a 0.45 μm filter (Millex^®^) to remove cell debris. The supernatant was treated with DNase I (Takara; 1:1000 dilution) in the presence of 5 mM MgCl_2_ for 30 min at 37°C to degrade extra-virion DNA. The reaction was stopped by adding 10 mM EDTA. Virion particles were precipitated with 30% PEG8000 (PEG8000; Wako Pure Chemical Industries; w/v) stock solution (final concentration, 6%) and the virion pellet was dissolved in 10 mM Tris–HCl (pH 7.6), 5 mM EDTA, and 0.5% SDS. Proteinase K (Roche, Switzerland) at 0.2 mg/ml was added and incubated at 56°C for at least 3 h. Then the samples were spun down briefly, and RNase A was added at 0.1 mg/ml (Roche’s RNase) and incubated for 30 min at 65°C. Salmon sperm DNA was added (15 μg/mL) as a carrier, and then the KSHV DNA was isolated by phenol chloroform isopropanol extraction followed by ethanol precipitation and rinsing with 70% ethanol, dried, and finally dissolved in 50 μL Tris-EDTA buffer (TE; 10 mM Tris [pH:8.0] and 1 mM EDTA). For the cellular DNA preparation, cells were washed with PBS and centrifuged in an Eppendorf tube at 7200 rpm for 5 min at 4°C. The pellet was gently suspended in 10 mM Tris–HCl (pH 7.6), 5 mM EDTA, and 0.5% SDS. The total DNA was extracted as a virus DNA preparation as described above without adding carrier DNA. The purified and dried DNA was dissolved in TE buffer, completely. The intracellular and the extracellular virion-associated KSHV DNA was quantified by amplification of KSHV using a specific primer set (each at 0.5 μM) of P8FW1 (75924-75946; GenBank: GQ994935.1): 5′-AGGCCGTCTCCCTAAACGGGACC-3′ and P8RV1 (76102-76080; GenBank: GQ994935.1): 5′-TTGTGTCA TAAAAGTCACGTGGG-3′. The quantification was carried out by using SYBR^®^ Green Master Mix (Thermo Fisher Scientific, United States) with a qPCR machine following the manufacturer’s guidelines (QuantStudio^TM^ 6 Flex, Thermo Fisher Scientific, United States). Triplicate reactions were performed and the average values were calculated according to the standard line. The intracellular KSHV DNA quantity was determined according to the standard which was the BAC36 DNA (Zhou et al., 2002).

### Statistical Analysis

Each experiment was performed at least three times and the results were reported as the mean ± standard error (SE) of the mean. A two tailed unpaired Student’s *t*-test was conducted in applicable cases to determine the statistical significance level, and probability values of *p* < 0.05 were considered statistically significant.

## Results

### Establishment of a Stable PML-Knockout PEL Cell Line

**FIGURE 1:**
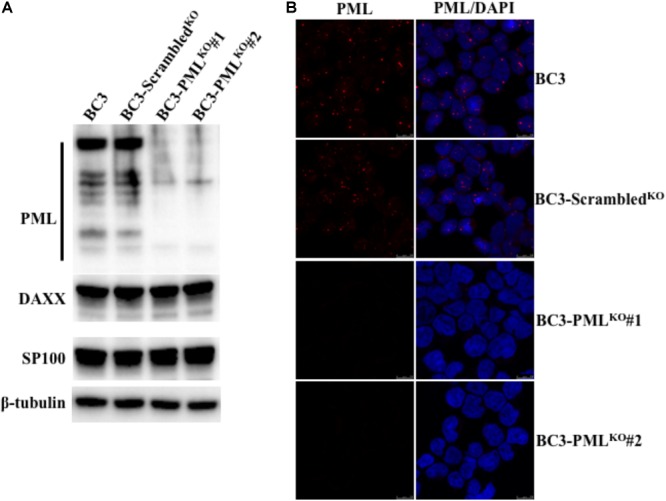
Establishment of stable PML-knockout BC3 cells. Stable PML-knockout BC3 cells (BC3-PML^KO^#1 and BC3-PML^KO^#2) and the control one (BC3-Scrambled^KO^) were generated from wild-type BC3 cells by puromycin selection after transduction with lentivirus as described in the Materials and Methods. **(A)** Western blot analysis of PML expression. Extracted total lysates from wild-type, control, and PML-knockout cells were separated on an SDS–PAGE and immunoblotted with antibodies to PML, DAXX and SP100. Specific proteins were visualized by using HRP conjugated secondary Abs. β-tubulin was used as a loading control. **(B)** Immunofluorescence analysis. The cells were fixed, permeabilized and stained with a mouse anti-PML Ab followed by an Alexa Fluor^®^ 546 conjugated anti-mouse IgG (red). The cell nuclei were stained with DAPI (blue).

Promyelocytic leukemia was localized both in the nucleo-cytoplasmic and chromatin fraction in TPA-induced BC3 cells (KSHV-infected), but only in the nucleo-cytoplasmic fraction in KSHV-uninfected BJAB cells (Ohsaki and Ueda, 2015). To investigate the functional involvement of PML in KSHV replication in greater detail, we first established stable PML-knockout BC3 cell lines with a CRISPR/Cas9 system. We isolated two monoclonal lines showing almost complete PML knockout by limited dilution after transducing the cells with lentivirus encoding CRISPR/Cas9 PML. We confirmed the PML knockout by Western blot and IFA analysis (**Figures 1A,B**). We also investigated the expression of DAXX and SP100, components of the PML-NB in PML-knockout BC3 cells by Western blot and IFA (**Figure 1A** and **Supplementary Figures S1A,B**). The expressions of DAXX and SP100 in PML-knockout cells showed no difference by Western blot analysis (**Figures 1A**). By IFA, some cells still showed DAXX and SP100 dots in the nucleus (**Supplementary Figures S1A,B**), despite the previous finding showed that Daxx and SP100 failed to accumulate in the NBs in the absence of PML (Zhong et al., 2000a). PML-knockout cells exhibited no significant change in growth rate or morphology compared to non-transduced or control cells (data not shown).

### PML Knockout Does Not Interfere With KSHV Latency or Episome Copy Numbers

**FIGURE 2:**
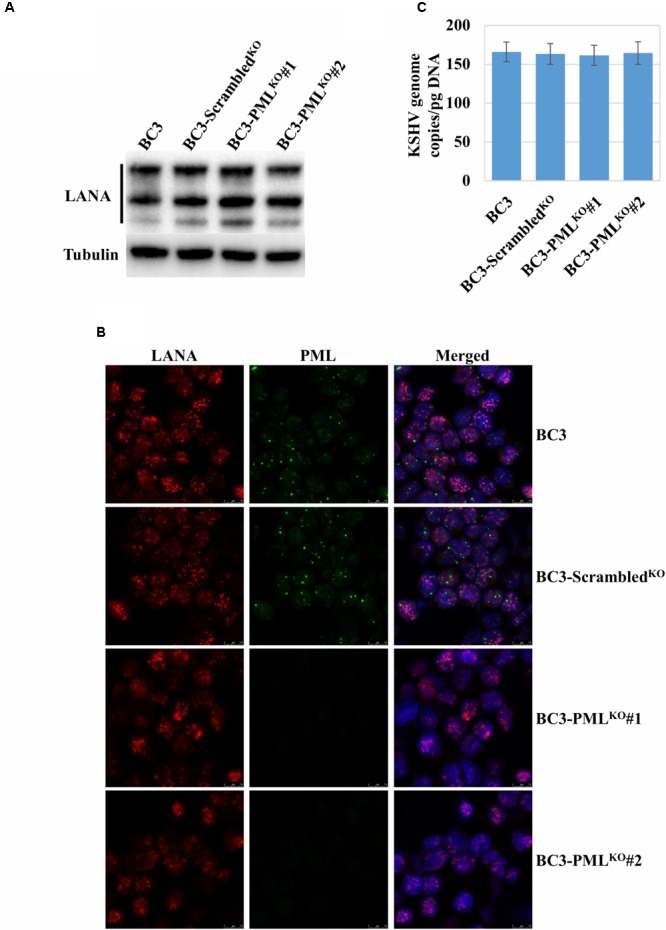
Analysis of LANA expression and KSHV episomes in the PML-knockout cells. **(A)** Immunoblot analysis of LANA. Total proteins were extracted from the wild-type (BC3), the control (BC3-Scrambled^KO^), and the stable PML-knockout cells (BC3-PML^KO^#1 and BC3-PML^KO^#2). The proteins separated on an SDS–PAGE were immunoblotted with a LANA antibody. These samples were the same as those shown **Figure 1A** and thus, the β-tubulin as a loading control for each lane was also the same as one in **Figure 1A**. **(B)** Immunofluorescence analysis of LANA and PML. The cells were stained with a rat anti-LANA and a mouse anti-PML antibody followed by Alexa Fluor^®^ 546 and Alexa Fluor^®^ 488 conjugated anti-rat IgG (red) and anti-mouse IgG (green), respectively. The cell nuclei were stained with DAPI and shown in blue. **(C)** KSHV episome copy number quantification. Total DNA from the cells was extracted and the KSHV episome copy number was quantified by qPCR with a KSHV gene-specific primer set and the data were shown KSHV genome copy/ pg DNA. The results are presented as an average of three replicates with error bars representing SEs.

In a previous study, PML did not interfere with the establishment of KSHV latency in iSLK cells artificially infected by recombinant KSHV (Gunther et al., 2014). PEL cells naturally infected with KSHV, maintain a stable number of KSHV episomes per cell dependent on the PEL cell types in patients (Uppal et al., 2014). Therefore, we asked whether PML knockout had any effect on the maintenance of KSHV latency and episome copy numbers. We analyzed the expression of LANA by Western blot and found that its expression level was similar among wild-type, control, and PML-knockout BC3 cells (**Figure 2A**). IFA staining with a LANA-specific Ab also showed a similar LANA dot pattern (**Figure 2B**) and the KSHV episome copies per cell by qPCR were not changed (**Figure 2C**). These results revealed that the absence of PML in KSHV-infected BC3 cells also did not interfere with the latency, episome copy numbers or expression of LANA.

### Induction of KSHV Lytic Replication in Wild-Type, Control, and PML-Knockout PEL Cells

**FIGURE 3:**
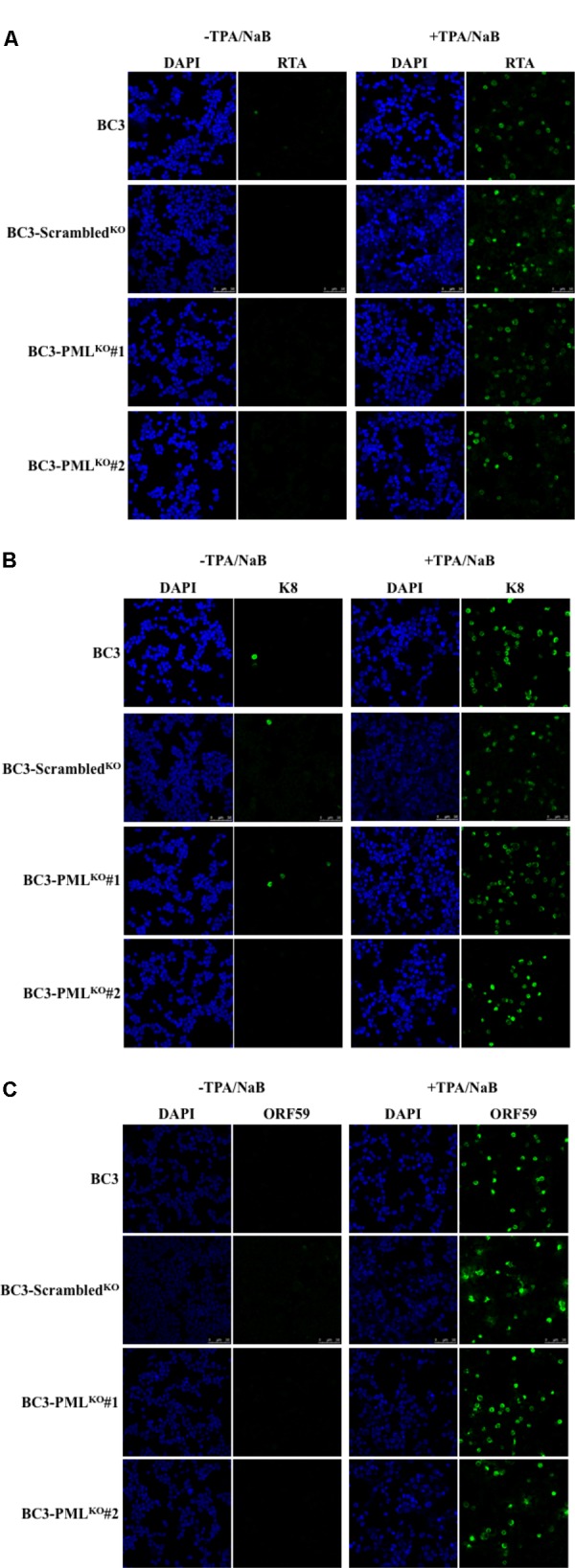
Induction of KSHV lytic replication by TPA and NaB treatment. The cells were treated with TPA (25 ng/mL) and NaB (0.6 mM) and incubated for 48 h. The collected cells were then stained with antibodies against the indicated specific proteins followed by an Alexa Fluor^®^ 488 conjugated IgG (green) and analyzed by a confocal microscope. Different panels show the staining of RTA **(A)**, K8 (K-bZIP) **(B)**, and ORF59 **(C)** with or without TPA/NaB treatment. The cell nuclei were stained with DAPI (blue). The experiment was performed at least three times independently and one representative result is shown.

Tetradecanoyl phorbol acetate (25 ng/mL) and NaB (0.3 mM) were used to activate entire KSHV lytic replication followed by production of progeny virion particles in stable KSHV-infected PEL cell lines (Ueda et al., 2002). The number of RTA-expressing cells at 48 h post-lytic induction appeared to be comparable among the wild-type, control, and PML-knockout cells and in K8 (K-bZIP) as well (**Figures 3A,B**). ORF59, a replication processivity factor (Lin et al., 1998), also showed comparable expression in PML-knockout, wild-type, and control BC3 cells (**Figure 3C**). Similar results were obtained at 24 and 72 h post-lytic induction (data not shown). Taken together, the results suggested that the early phase of KSHV lytic reactivation was not dependent on endogenous PML.

### Expression Dynamics of KSHV Immediate-Early (IE) and Early (E) Proteins

**FIGURE 4:**
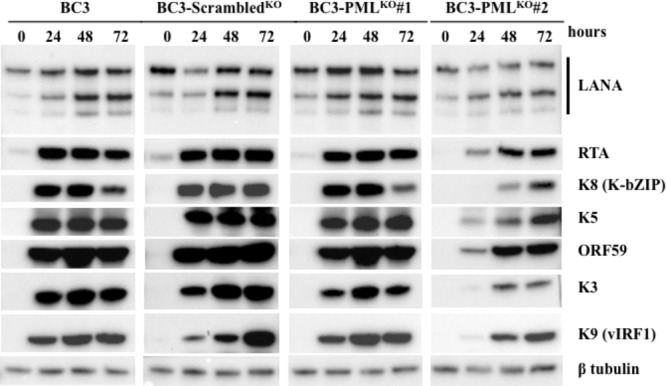
Expression dynamics of KSHV lytic replication-related proteins. The KSHV lytic replication in wild-type (BC3), control (BC3-Scrambled^KO^), and stable PML-knockout cells (BC3-PML^KO^#1 and BC3-PML^KO^#2) was induced with the treatment of TPA (25 ng/mL) and NaB (0.3 mM) and the cells were collected at the indicated time points. The total proteins were extracted and immunoblotted with the antibodies against the indicated specific proteins. β-tubulin was used as a loading control. The experiment was performed at least three times independently and one representative result is shown.

The master regulator of lytic reactivation from KSHV latency, replication, and transcription activator (RTA) can induce the entire cascade of lytic gene expression in PEL cells followed by successful DNA replication and release of virion particles (Gradoville et al., 2000). Chemical induction for KSHV lytic replication with TPA and/or NaB leads to expression of RTA in 4–6 h, followed by the expression of K3, K5, K9 (vIRF1), ORF59, and K8 (K-bZIP) (Gradoville et al., 2000; Okuno et al., 2002). Therefore, we tested the expression dynamics of such an immediate-early protein, RTA, and of early proteins such as K3, K5, K9 (vIRF1), ORF59, and K8 (K-bZIP) in PML-knockout BC3 cell lines. As shown in **Figure 4**, the expression levels and kinetics of RTA and other early proteins were unchanged at each time point in PML-knockout (BC3-PML^KO^#1), control, and wild-type cells (BC3), though BC3-PML^KO^#2 showed a slightly lower expression level of the proteins. These facts suggested that endogenous PML should have no effect on the expression of such KSHV IE and E genes. Moreover, the expression dynamics of cellular PML, DAXX, and SP100 after lytic induction were also comparable among PML-knockout, control, and wild-type cells (**Supplementary Figure S2**).

### KSHV DNA Replication, Virion Production, and Late Gene Expressions in PML-Knockout BC3 Cells

**FIGURE 5:**
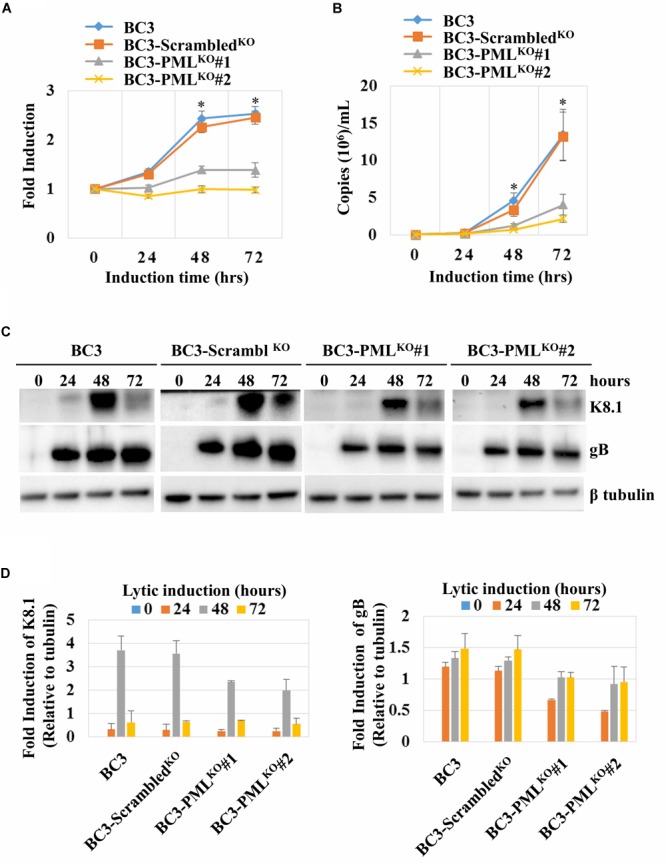
Kaposi’s sarcoma-associated herpesvirus lytic DNA replication, virion production, and late-gene expression kinetics in PML-knockout cells. KHSV lytic replication was induced with TPA (25 ng/mL) and NaB (0.6 mM) treatment and the cells and the supernatants were collected at the indicated time points. The total DNA from the cells and KSHV virion DNA from the concentrated culture supernatants were extracted and the viral DNA was quantified as described in the Materials and Methods. **(A)** Intracellular KSHV DNA replication normalized by an endogenous control gene *GAPDH.* The data were shown as fold induction where the KHSV genomic DNA copy at the time zero was set at 1. **(B)** Extracellular virion production. The data were presented as an average of three replicates with error bars representing SEs. ^∗^Statistically significant; *P* < 0.05. **(C)** Total proteins were extracted from the induced cells and immunoblotted with the antibodies against the indicated specific protein. β-tubulin was used as a loading control. **(D)** Graphs show relative expression levels of K8.1 and gB to β-tubulin generated from the Western blot band intensities.

A previous report showed that another herpesvirus, Epstein-Barr virus (EBV), formed a replication compartment adjacent to the PML-NB during its lytic DNA replication (Amon et al., 2006). On the other hand, PML-NBs might play a role in post-replication DNA processing by linking with the sites of viral DNA synthesis (Jul-Larsen et al., 2004). In terms of lytic replication, PML knockout downregulated KSHV intracellular DNA replication by a factor of one-third compared to wild-type cells or control cells (**Figure 5A**). The PML-knockout clone 2 (BC3-PML^KO^#2) seemed rather the lower replication level, probably because the expression of early proteins was slightly lower, as mentioned above (**Figure 4**). In addition, the KSHV virion production was decreased to one-fourth at 48 h and to one-sixth at 72 h of lytic induction in PML-knockout cells (**Figure 5B**). The lower DNA replication might lead to lower expression levels of KSHV late genes. Therefore, we next analyzed the expression kinetics of virion-associated glycoprotein K8.1 and gB, two late genes of KSHV by Western blot. As expected, the expression of both K8.1 and gB was moderately but surely decreased at 48 h of lytic induction in PML-deficient BC3 cells (**Figures 5C,D**).

### Exogenous PML Upregulated the KSHV Lytic DNA Replication, Virion Production, and Late Gene Expression

**FIGURE 6:**
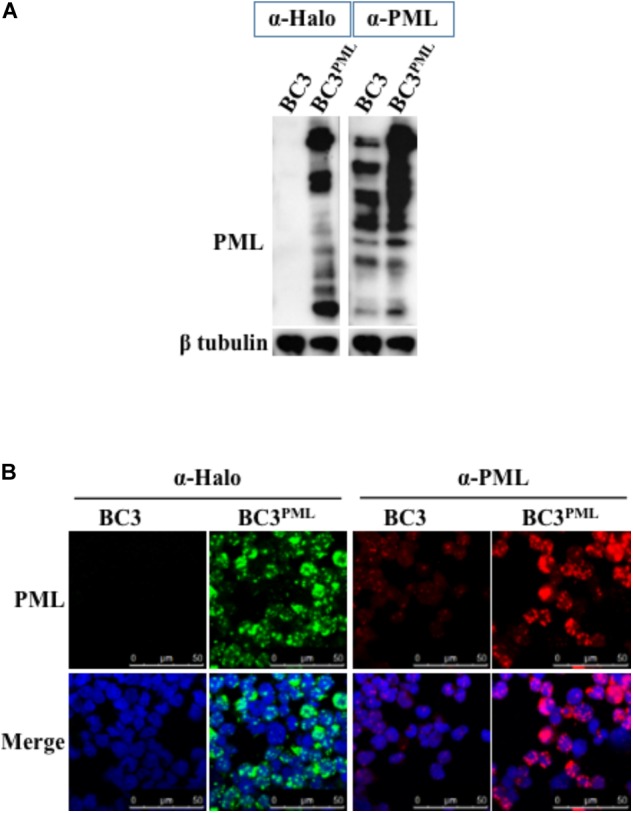
Overexpression of PML in BC3 cells. Wild-type BC3 cells were transduced with a halo-tagged PML-encoding retrovirus, and stable PML-expressing cells were established after hygromycin selection. **(A)** Total protein from the cells were extracted and immunoblotted with an anti (α)-Halo and an α-PML Ab for detection of PML. β-tubulin was used as a loading control. **(B)** Cells stably expressing PML were fixed and stained with a mouse α-Halo and a rabbit α-PML Ab and signals were visualized via immunofluorescence using an Alexa Fluor^®^ 488 (green) conjugated anti-mouse IgG and an Alexa Fluor^®^ 548 (red) conjugated anti-rabbit IgG, respectively. The cell nuclei were stained with DAPI (blue).

**FIGURE 7:**
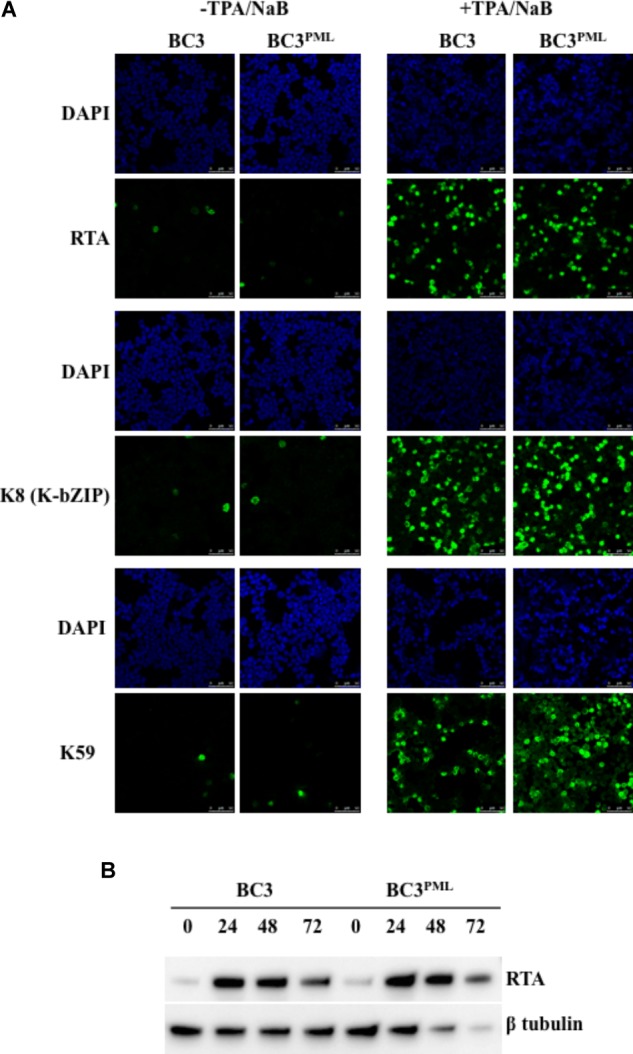
Induction of KSHV lytic replication in BC3 cells stably overexpressing PML. Wild-type and PML-overexpressing cells were treated with TPA (25 ng/mL) and NaB (0.6 mM) and incubated up to 72 h. **(A)** Immunofluorescence analysis. The cells at 0 or 48 h post-induction were collected and stained with antibodies against the indicated specific proteins followed by an Alexa Fluor^®^ 488 conjugated IgG (green) and analyzed by a confocal microscope. The cell nuclei were stained with DAPI (blue). **(B)** Western blot analysis. Total protein was extracted from the cells collected at the indicated time points and immunoblotted with the antibodies against the indicated specific proteins. β-tubulin was used as a loading control.

**FIGURE 8:**
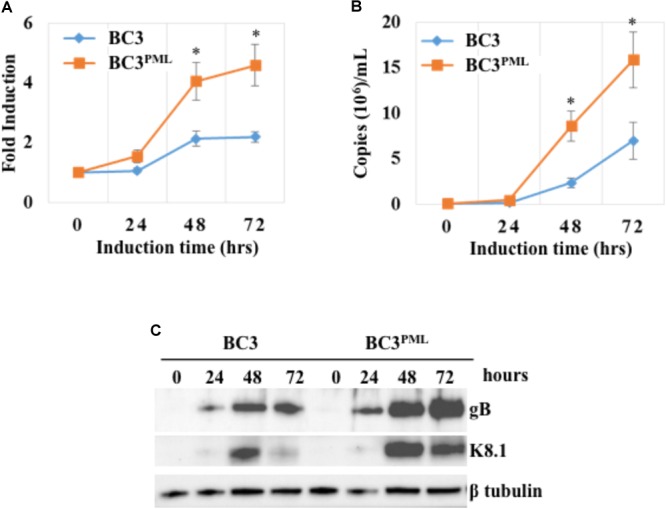
Kaposi’s sarcoma-associated herpesvirus lytic DNA replication, virion production, and late-gene expression were upregulated by exogenous PML. The wild-type and BC3 cells stably expressing exogenous PML were treated with TPA (25 ng/mL) and NaB (0.6 mM) to induce the KSHV lytic replication. The total DNA from the cells and KSHV virion DNA from the concentrated culture supernatants were extracted at the indicated time points. The viral DNA was quantified using a KSHV gene-specific primer set as described in the Materials and Methods. **(A)** Intracellular KSHV DNA, normalized by endogenous control gene *GAPDH.* **(B)** Extracellular virion associated DNA. The data are presented as an average fold induction of three replicates with error bars representing the SEs. ^∗^Statistically significant; *P* < 0.05. **(C)** Total protein was extracted from the induced cells and immunoblotted with the antibodies against the indicated specific proteins. β-tubulin was used as a loading control.

Promyelocytic leukemia knockout reduced the KSHV DNA replication and virion production in KSHV-infected BC3 cells (**Figure 5**). We speculated that PML overexpression would augment the DNA replication and virion production. To test this, PML was overexpressed in wild-type BC3 cells by retroviral transduction, and a stable cell line (BC3^PML^) was generated with hygromycin selection (**Figures 6A,B**). Exogenous PML enlarged the size of the PML dots without affecting DAXX or SP100 expression (**Supplementary Figures S3A–C**). There was no significant difference in RTA and/or K8 (KbZIP) expression by IFA between the wild-type (BC3) and PML-overexpressing cells (BC3^PML^) at 48 h after TPA/NaB treatment (**Figure 7A**). The expression kinetics of RTA was also comparable at different time points after lytic induction (**Figure 7B**). As expected, lytic DNA replication and virion production in the exogenous PML-expressing cells (BC3^PML^) was increased more than two folds at 48 and 72 h after lytic induction compared with wild-type cells (**Figures 8A,B**). Moreover, the expression of virion-associated KSHV late genes, K8.1 and gB, was also increased in PML-overexpressing cells (BC3^PML^) (**Figure 8C**).

### Co-localization of PML With KSHV Lytic Proteins and Their Distribution

**FIGURE 9:**
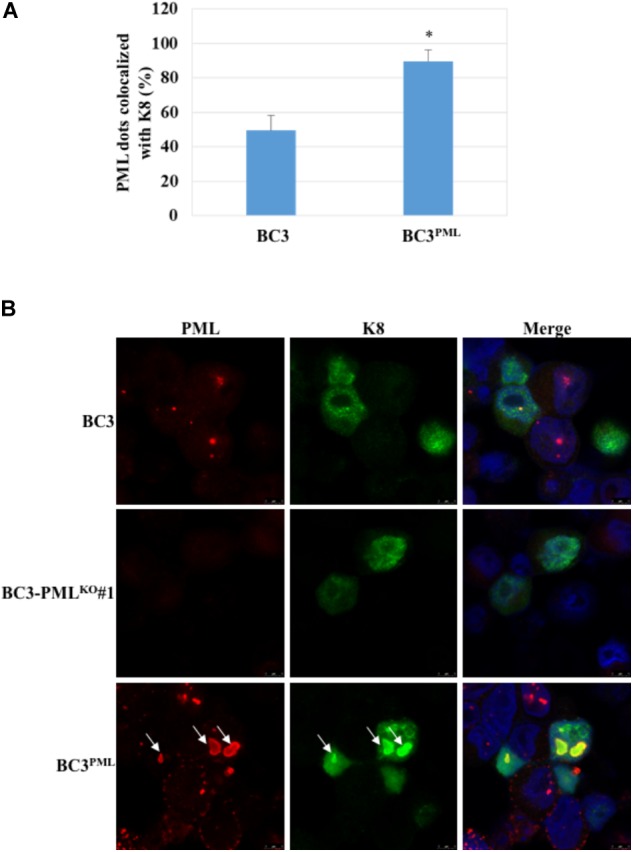
Subcellular distribution of K8 (K-bZIP) in wild-type, PML-knockout, and PML-overexpressing BC3 cells. The cells were collected 48 h after treatment with TPA (25 ng/mL) and NaB (0.6 mM). Then the cells were fixed and stained with an anti-K8 (K-bZIP) Ab (mouse) and an anti-PML (rabbit) followed by Alexa Fluor^®^ 488 conjugated mouse IgG (green) and an Alexa Fluor^®^ 548 conjugated rabbit IgG (red), respectively. The cell nuclei were highlighted with DAPI. **(A)** Percentage of PML dots colocalized with K8 (K-bZIP) in wild-type and PML-overexpressing BC3 cells. The results showed the percentages of K8 (K-bZIP) and PML colocalized dots. PML dots were counted in the lytic induced BC3 and BC3^PML^ cells. The data are presented as an average of three replicates with error bars representing SEs. ^∗^Statistically significant; *P* < 0.05. **(B)** K8 (K-bZIP) and PML colocalization pattern. Arrows indicate the accumulation of K8 (K-bZIP) to the PML dots.

It has been reported that PML was co-localized with several KSHV early lytic proteins, such as K8 (K-bZIP), K9 (vIRF-1), and a tegument protein encoded by ORF75 (Katano et al., 2001; Pozharskaya et al., 2004; Full et al., 2014). Here, we checked the colocalization of PML with K8 (K-bZIP) in TPA/NaB-induced BC3 cells. First, we quantified the PML/K-bZIP colocalization in wild-type BC3 cells, and then we checked the distribution of K8 (K-bZIP) in PML-knockout BC3 cells at 48 h after lytic induction. Basically, K8 (K-bZIP) was distributed diffusely throughout the nucleus, and not all K8 (K-bZIP) was colocalized with PML. Around 50% of PML dots were colocalized with K8 (K-bZIP) in wild-type BC3 cells (**Figures 9A,B**). The colocalization pattern was changed in PML-overexpressing cells (BC3^PML^), where PML/K-bZIP colocalization reached 80% (**Figures 9A,B**). In addition, exogenous PML made the PML-NBs much larger in size. Interestingly, we also found that additional K8 (K-bZIP) was accumulated in the PML-NBs as larger dots in the BC3^PML^ cells (**Figure 9B**). These results suggest that exogenous PML should recruit additional K8 (K-bZIP) to PML-NBs and support KSHV lytic replication.

## Discussion

Kaposi’s sarcoma-associated herpesvirus maintains dual replication phases in PEL cells: a non-productive episome maintaining latent cycle with limited viral gene expression and a lytic cycle with high expression of viral lytic genes followed by production of progeny viruses (Ye et al., 2011; Uppal et al., 2014). Following primary infection, the KSHV genome is epigenetically modified by host cellular factors to evade the host immune system and establishes KSHV latent infection as a default (Toth et al., 2010; Ye et al., 2011). Once RTA is activated, the viral lytic cycle is induced and several lytic gene products including RTA interact with many host cellular factors during the KSHV lytic replication (Liang et al., 2002; Ye et al., 2011). PML is one of these cellular proteins and interacts with KSHV proteins such as LANA, K8 (K-bZIP), K9 (vIRF1), and ORF75 (Katano et al., 2001; Pozharskaya et al., 2004; Marcos-Villar et al., 2009; Full et al., 2014). Though PML is reported to be important for host defense and exhibits antiviral activities (Geoffroy and Chelbi-Alix, 2011; Lunardi et al., 2011), a few studies have reported that PML might also be a key cellular factor for viral DNA processing and replication in some cases (Jul-Larsen et al., 2004; Chung and Tsai, 2009). Moreover, PML-NB has been shown to interact with many viral proteins, including herpesviruses, adenoviruses, and papillomaviruses (Maul et al., 1993; Szekely et al., 1993; Carvalho et al., 1995; Ahn et al., 1998; Day et al., 1998; Adamson and Kenney, 2001; Everett, 2001).

Promyelocytic leukemia-nuclear bodies might serve as a site for DNA transcription, replication, and DNA repair, possibly by recruiting p53 (Bischof et al., 2001; Carbone et al., 2002; Dellaire and Bazett-Jones, 2004). As for SV40, PML might also be a positive regulator of the viral replication, based on the pervious report in which attenuation of origin-dependent viral replication was observed in PML-depleted cells (Boichuk et al., 2011). Jul-Larsen et al. (2004) reported that PML-NBs functioned as the sites of SV40 and BKV viral DNA synthesis, thereby playing a role in post-replication DNA processing, and other reports showed several DNA viral genome replicates in the vicinity of PML-NBs (Maul, 1998; Everett, 2001; Jul-Larsen et al., 2004). The HCMV genome is located near PML-NBs with transcriptional activity, which should also help in viral DNA replication (Ishov et al., 1997; Saffert and Kalejta, 2008). The EBV genome forms a collective replication compartment adjacent to PML-NBs during the lytic cycle (Amon et al., 2006). In addition, recent studies reported that PML plays both adventitious and harmful roles in HSV-1 replication, and that the HSV viral yield is significantly lower in PML-knockout cells with low multiplicity of infection (Xu et al., 2016; Xu and Roizman, 2017). Therefore, it is speculated that PML might also be important for KSHV lytic DNA replication.

As for KSHV, the influence of PML on the viral latent and lytic replication is still obscure and controversial (Maul, 1998; Everett, 2001). In this study, we tried to uncover the functional influence of PML on KSHV replication in BC3 cells in detail and we found that PML positively regulated the KSHV lytic DNA replication, virion production, and late gene expression. Moreover, exogenous PML recruited additional K8 (K-bZIP) to the PML-NB bodies and PML/K-bZIP colocalization dots also increased in the presence of PML. On the other hand, KSHV latency in PEL cells was not affected either by the absence of PML or the presence of exogenous PML, which appears to agree with a report by Gunther et al. (2014), in which depletion of PML-NB components was shown not to interfere with the establishment of KSHV latency during primary infection both in EA hy926 and SLK cells (Gunther et al., 2014).

Promyelocytic leukemia acts both as a transcriptional co-activator and a co-repressor with different kinds of cellular proteins (Zhong et al., 2000b). In addition, PML has been shown to interact with several viral proteins, including KSHV K8 (K-bZIP), K9 (vIRF-1), and ORF75 (Katano et al., 2001; Pozharskaya et al., 2004; Full et al., 2014). K9 (vIRF-1) binds with p53, p300 and CREB and K8 (K-bZIP) binds with p53, and these cellular proteins are found in the PML bodies (Li et al., 2000; Seo et al., 2000, 2001; Katano et al., 2001; Nakamura et al., 2001; Wu et al., 2001). CREB acts as a transcription factor, whereas p300 regulates transcription as a histone acetyltransferase with some regulatory effect on p53 (Lonze and Ginty, 2002; MacPartlin et al., 2005; Wen et al., 2010). We expected that PML along with p53 or p300 or CREB might have some regulatory effect, at least on the expression dynamics of K8 (K-bZIP) and K9 (vIRF-1). However, neither the absence of PML nor the presence of exogenous PML affected the expression dynamics of immediate-early and early genes, including K8 (K-bZIP) and K9 (vIRF-1).

On the other hand, KSHV intracellular lytic DNA replication was significantly impaired in the PML-deficient cells, especially in the later stage of the lytic phase (at 48 and 72 h of induction) (**Figure 5A**). Moreover, the extracellular virion production was also impaired in PML-deficient cell culture supernatants, which confirmed the downregulation of lytic DNA replication (**Figure 5B**). Accordingly, intracellular lytic DNA replication and extracellular virion production were augmented by exogenous PML in BC3 cells (**Figures 8A,B**). Increase or decrease of the lytic DNA replication and/or virion production were further confirmed by concomitant expression of late genes such as K8.1 and gB by Western blot analysis (**Figures 5C, 8C**). To examine this point, we tried complementation of PML-KO cells. However, the impairments in KSHV lytic replication and progeny virus production were not reversed by the complementation, though it is not clear why the complementation did not work and PML-KO could lead to irreversible state. In the case of HSV-1, PML knockout was also tried and the virus production was decreased (Xu et al., 2016; Xu and Roizman, 2017). However, in these reports, there were no experiment on PML complementation, and thus the effect of PML knockout could be externally established and not easily recovered due to unknown reasons.

Lefort and Flamand reported that K8 (K-bZIP) was necessary for KSHV lytic DNA replication in BC3 and BCBL-1 cells (Lefort and Flamand, 2009). They found a severe decrease in DNA replication and virion production in the K8 (K-bZIP)-knockdown BC3 and BCBl-1 cells. As K8 (K-bZIP) was reported to be a component of the replication compartment and the pre-replication complex recruited to ori-Lyt, it was suggested to be important for KSHV DNA replication (Wang et al., 2006). As discussed above, K8 (K-bZIP) colocalized with PML during KSHV lytic replication in the present study. We found that 50% of the PML dots were colocalized with K8 (K-bZIP) at 48 h after lytic induction, whereas the number of PML/K-bZIP-colocalized dots was significantly increased (∼80%) in PML-overexpressing cells (**Figure 9B**). PML-NBs became larger in size with exogenous PML in BC3 cells and interestingly the additional K8 (K-bZIP) seemed to be accumulated with the dots in the PML-overexpressing cells (**Figure 9A**). On the other hand, PML-NBs act as an effector of DNA damage, and the number of PML-NBs and expression level of PML increase in response to double-stranded DNA breaks (Xu et al., 2003; Dellaire et al., 2006). K8 (K-bZIP) binds with p53 to permit its own recruitment into PML-NBs, and p53 can activate DNA repair proteins during sustained DNA damage (Katano et al., 2001; Meek, 2009; Zhang et al., 2012). PML is also linked to the DNA damage response during hepatitis B virus (HBV) replication, and viral DNA replication was downregulated by PML suppression without affecting the amount of secreted HBsAg and HBeAg in culture medium (Chung and Tsai, 2009).

Taken together, our results suggest that PML should act as a positive regulator of KSHV lytic DNA replication, probably via one or two different mechanisms. First, PML appears to act on KSHV DNA replication indirectly by recruiting K8 (K-bZIP) to the PML-NBs. In this case, K8 (K-bZIP) goes through PML-NBs for its proper function in KSHV DNA replication, and K8 (K-bZIP) might be sumoylated at PML-NBs (Izumiya et al., 2013). Second, PML could positively regulate KSHV lytic DNA replication directly through the DNA repair signaling pathway.

## Author Contributions

MH preformed almost all experiments including data collection described in the manuscript, summarized data, and prepared the manuscript. EO and TH instructed the experiments and advised how to do the experiments. KU directed the experiments and also instructed the experiments.

## Conflict of Interest Statement

The authors declare that the research was conducted in the absence of any commercial or financial relationships that could be construed as a potential conflict of interest.

## Supplementary Material

FIGURE S1 Detection of PML-NB components, DAXX and SP100 in PML-knockout BC3 cells. Cells were fixed and stained with the indicated Abs followed by an Alexa Fluor^®^ 488 conjugated or an Alexa Fluor^®^ 548 conjugated IgG. The cell nuclei were stained with DAPI. **(A)** Staining of DAXX and PML. **(B)** Staining of SP100 and PML.

FIGURE S2 Expression kinetics of PML-NB components in KSHV lytic induced cells. Cells were collected at the indicated time points after treatment with TPA (25 ng/mL) and NaB (0.6 mM) to induce the KSHV lytic replication. Total protein was extracted and immunoblotted with the specific Abs against the indicated cellular proteins. β-tubulin was used as a loading control.

FIGURE S3 Detection of PML-NB components, DAXX and SP100 in PML-overexpressed BC3 cells. Wild-type BC3 cells were transduced with a halo-tagged PML-encoding retrovirus, and stable PML-expressing cells were established after hygromycin selection. **(A)** Total protein from the cells were extracted and immunoblotted with an α-DAXX and an α-SP100 Ab. β-tubulin was used as a loading control. Cells were fixed and stained with the indicated Abs followed by Alexa Fluor^®^ 488 conjugated or Alexa Fluor^®^ 548 conjugated IgG. The cell nuclei were stained with DAPI. **(B)** Staining of DAXX (red) and PML (green). **(C)** Staining of SP100 (red) and PML (green).
