# Supplementary figures and images for: Importance of Promyelocytic Leukema Protein (PML) for Kaposi’s Sarcoma-Associated Herpesvirus Lytic Replication
Source: Front Microbiol. 2018 Oct 8;9:2324. doi: 10.3389/fmicb.2018.02324 (PMC6186782; doi:10.3389/fmicb.2018.02324)

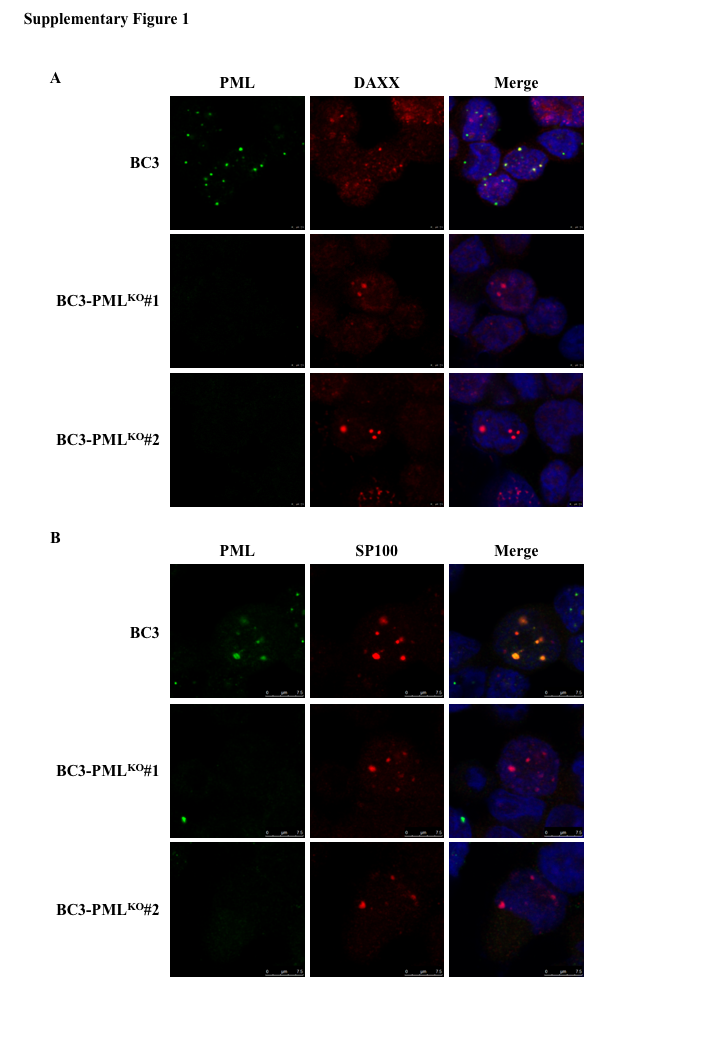

Supplement: FIGURE S1 — Detection of PML-NB components, DAXX and SP100 in PML-knockout BC3 cells. Cells were fixed and stained with the indicated Abs followed by an Alexa Fluor® 488 conjugated or an Alexa Fluor® 548 conjugated IgG. The cell nuclei were stained with DAPI. (A) Staining of DAXX and PML. (B) Staining of SP100 and PML. [file Image_1.TIFF]

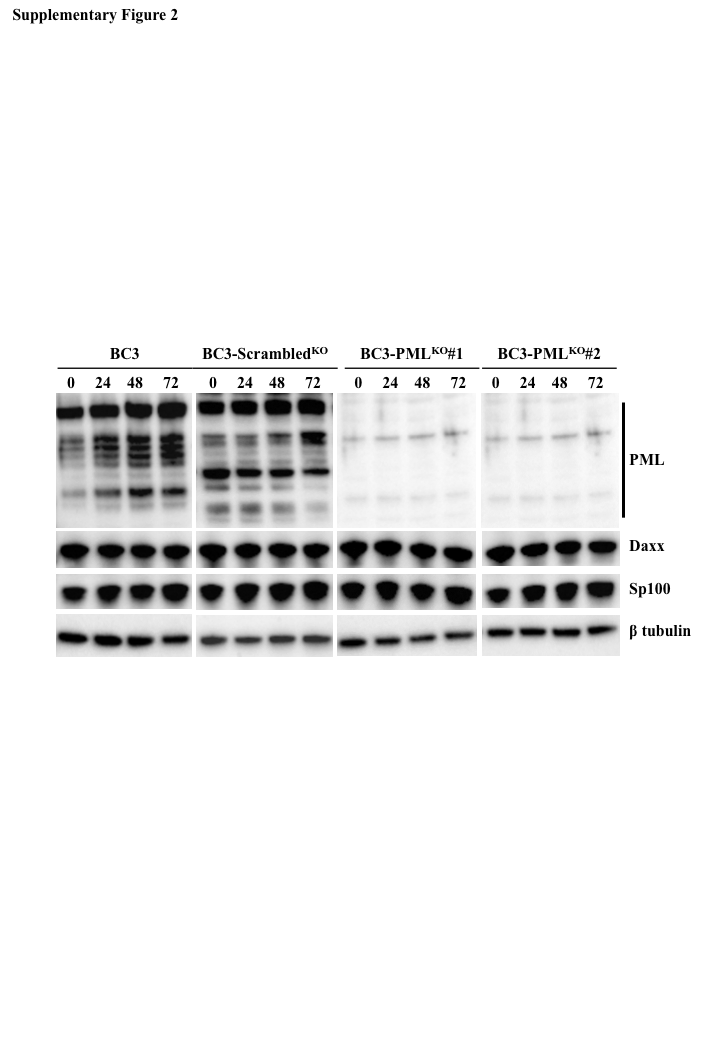

Supplement: FIGURE S2 — Expression kinetics of PML-NB components in KSHV lytic induced cells. Cells were collected at the indicated time points after treatment with TPA (25 ng/mL) and NaB (0.6 mM) to induce the KSHV lytic replication. Total protein was extracted and immunoblotted with the specific Abs against the indicated cellular proteins. β-tubulin was used as a loading control. [file Image_2.TIFF]

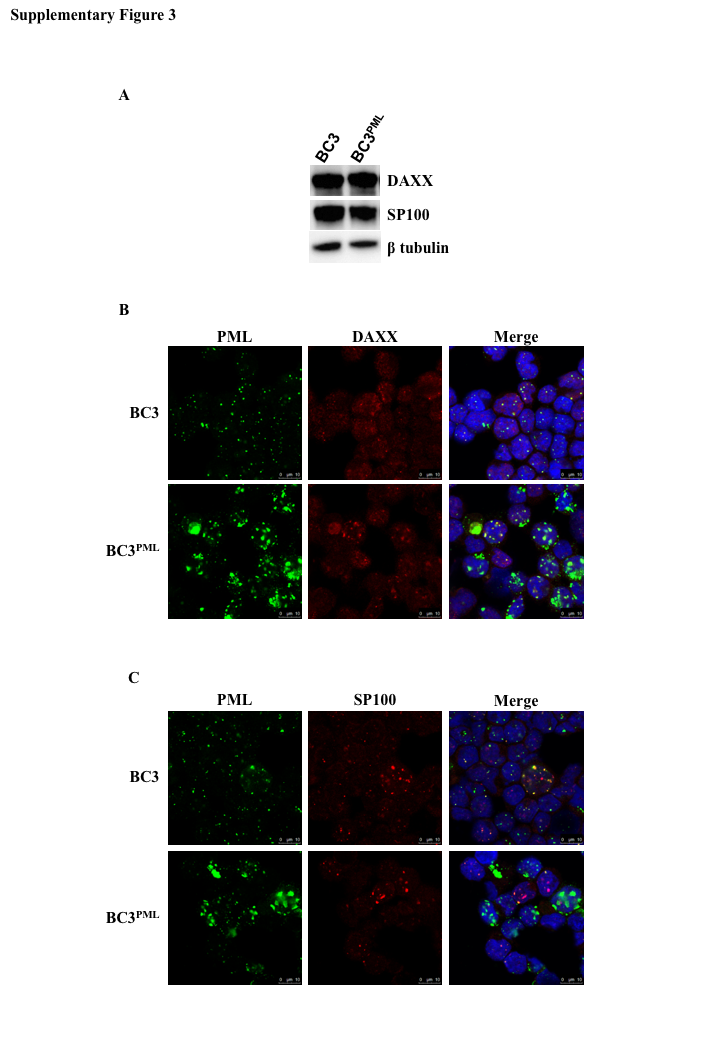

Supplement: FIGURE S3 — Detection of PML-NB components, DAXX and SP100 in PML-overexpressed BC3 cells. Wild-type BC3 cells were transduced with a halo-tagged PML-encoding retrovirus, and stable PML-expressing cells were established after hygromycin selection. (A) Total protein from the cells were extracted and immunoblotted with an α-DAXX and an α-SP100 Ab. β-tubulin was used as a loading control. Cells were fixed and stained with the indicated Abs followed by Alexa Fluor® 488 conjugated or Alexa Fluor® 548 conjugated IgG. The cell nuclei were stained with DAPI. (B) Staining of DAXX (red) and PML (green). (C) Staining of SP100 (red) and PML (green). [file Image_3.TIFF]
